# Ribozyme-mediated CRISPR/Cas9 gene editing in pyrethrum (*Tanacetum cinerariifolium*) hairy roots using a RNA polymerase II-dependent promoter

**DOI:** 10.1186/s13007-022-00863-5

**Published:** 2022-03-16

**Authors:** Jia-Wen Li, Tuo Zeng, Zhi-Zhuo Xu, Jin-Jin Li, Hao Hu, Qin Yu, Li Zhou, Ri-Ru Zheng, Jing Luo, Cai-Yun Wang

**Affiliations:** 1 Key Laboratory for Biology of Horticultural Plants, Ministry of Education, Huazhong Agricultural University, Wuhan, 430070 China; 2 School of Life Sciences, Guizhou Normal University, Guiyang, 550025 China

**Keywords:** CRISPR/Cas9, Hairy roots, Ribozyme, *Tanacetum cinerariifolium*

## Abstract

**Background:**

Traditional CRISPR/Cas9 systems that rely on *U6* or *U3* snRNA promoters (RNA polymerase III-dependent promoters) can only achieve constitutive gene editing in plants, hampering the functional analysis of specifically expressed genes. Ribozyme-mediated CRISPR/Cas9 systems increase the types of promoters which can be used to transcribe sgRNA. Therefore, such systems allow specific gene editing; for example, transcription of the artificial gene Ribozyme-sgRNA-Ribozyme (*RGR*) is initiated by an RNA polymerase II-dependent promoter. Genetic transformation is indispensable for editing plant genes. In certain plant species, including pyrethrum, genetic transformation remains challenging to do, limiting the functional verification of novel CRISPR/Cas9 systems. Thus, this study’s aim was to develop a simple *Agrobacterium rhizogenes*-mediated hairy root transformation system to analyze the function of a ribozyme-mediated CRISPR/Cas9 system in pyrethrum.

**Results:**

A hairy root transformation system for pyrethrum is described, with a mean transformation frequency of 7%. Transgenic hairy roots transformed with the pBI121 vector exhibited significantly increased beta-glucuronidase staining as a visual marker of transgene expression. Further, a ribozyme-based CRISPR/Cas9 vector was constructed to edit the *TcEbFS* gene, which catalyzes synthesis of the defense-related compound (E)-β-farnesene in pyrethrum. The vector was transferred into the hairy roots of pyrethrum and two stably transformed hairy root transgenic lines obtained. Editing of the *TcEbFS* gene in the hairy roots was evaluated by gene sequencing, demonstrating that both hairy root transgenic lines had DNA base loss at the editing target site. Gas chromatography–mass spectrometry showed that the (E)-β-farnesene content was significantly decreased in both hairy root transgenic lines compared with the empty vector control group. Altogether, these results show that *RGR* can be driven by the *CaMV35S* promoter to realize *TcEbFS* gene editing in pyrethrum hairy roots.

**Conclusion:**

An *A. rhizogenes*-mediated hairy root transformation and ribozyme-mediated CRISPR/Cas9 gene editing system in pyrethrum was established, thereby facilitating gene editing in specific organs or at a particular developmental stage in future pyrethrum research.

**Supplementary Information:**

The online version contains supplementary material available at 10.1186/s13007-022-00863-5.

## Background

CRISPR systems can modify target genes in plants by substituting, removing, inserting, or knocking out DNA [1]. In these systems, a single guide RNA (sgRNA) forms a complex with the Cas9 protein and guides the Cas9 protein to bind to a specific target DNA for gene editing [2]. Since the first successful application of a CRISPR system [3], this technology has been continuously refined and optimized to improve editing efficiency and accuracy [4, 5]. In recent years, CRISPR systems have been successfully applied to food crops, such as maize (*Zea mays* L.) [6]; cash crops, such as cotton (*Gossypium hirsutum*) [7]; fruits, including strawberry (*Fragaria ananassa*) [8]; and medicinal crops, including Chinese wolfberry (*Lycium ruthenicum*) [9], and is considered a new and effective tool for use in plant gene function research [10].

In the CRISPR/Cas9 system, the transcription of sgRNA is key. Transcription in eukaryotes is conducted by three RNA polymerase enzymes [11]: RNA polymerase I transcribes large rRNAs; RNA polymerase II synthesizes mRNAs; and RNA polymerase III transcribes small noncoding RNAs, such as the U6 snRNA [12]. Accordingly, their corresponding promoters were also divided into three types, namely RNA polymerase I-dependent promoter (pol I promoter), RNA polymerase II-dependent promoter (pol II promoter) and RNA polymerase III-dependent promoter (pol III promoter) [13–15]. Specific gene silencing requires specific promoters [16]; however, specific promoters are usually driven by RNA polymerase II and cannot be directly used for sgRNA transcription because the RNA undergoes extensive processing and modification at both ends to become a mRNA [17]. Previous researchers have used a root-cap-specific promoter or stomatal lineage-specific promoter (both are pol II promoters) to initiate *Cas9* mRNA transcription, with the *AtU6-26* promoter (a pol III promoter belonging to non-tissue specific constitutive promoters [17]) used to initiate sgRNA transcription, and thereby achieved tissue-specific gene editing in Arabidopsis [18]. Yet the mRNA can be transported long distances to other tissues [19], and constitutive expression of sgRNA, driven by the *U6* or *U3* snRNA promoters, may bind to the Cas9 protein outside of the specific tissue of interest, resulting in gene editing occurring in unintended locations.

The development of the ribozyme-mediated CRISPR/Cas9 system represents a remarkable improvement, because it can theoretically enhance gene editing’s specificity by using the pol II promoter. The common feature of this system is that ribozyme sequences, such as the hammerhead-type ribozyme (HH) [20] or hepatitis delta virus ribozyme (HDV) [21], are added at the ends of an sgRNA sequence, and when transcribed into RNA, the covalent bond of RNA is broken via the self-cleavage property of such ribozymes, with additional structures at both ends of RNA separated with ribozyme. In the first report [17], an ADH1 promoter (a pol II promoter of yeast) was used to drive the expression of a ribozyme-sgRNA-ribozyme (RGR) artificial gene, and an sgRNA was generated by ribozyme self-cleavage, facilitating site-specific gene editing.

Pyrethrum (*Tanacetum cinerariifolium*) is a perennial herbaceous plant belonging to the Asteraceae family that has been cultivated for centuries to extract botanical insecticides from its dried flowers [22]. (E)-β-farnesene is a volatile organic compound expressed in different tissues of pyrethrum that is considered to be related to insect resistance [23–25]. Although the (E)-β-farnesene synthase gene (*TcEbFS*) has been identified, and (E)-β-farnesene synthase expressed in vitro can catalyze (E)-β-farnesene synthesis [24], it has been postulated that *TcEbFS* has diverse functions in different pyrethrum tissues [23–25]. Therefore, further characterization of *TcEbFS* function and tissue-specific gene editing in pyrethrum is desirable.

To conduct gene editing research, an efficient transformation system is essential; however, no transformation system suitable for pyrethrum has yet been developed. *Agrobacterium rhizogenes*-mediated hairy root transformation is considered a rapid, simple, and highly efficient method that enables rapid characterization of improved CRISPR/Cas9 systems in hairy roots [26–29]. This report describes an *A. rhizogenes*-mediated hairy root transformation system that can quickly verify the effect of gene editing in pyrethrum, and tests the gene editing effect of a ribozyme-mediated CRISPR/Cas9 system using a pol II promoter in this context. Compared with traditional gene editing methods that rely on *U6* or *U3* snRNA promoters, this versatile method has unique advantages for use in pyrethrum breeding and gene function research that could be applied in future studies.

## Results

### A strategy for *A. rhizogenes*-mediated hairy root transformation system in pyrethrum

**Fig. 1:**
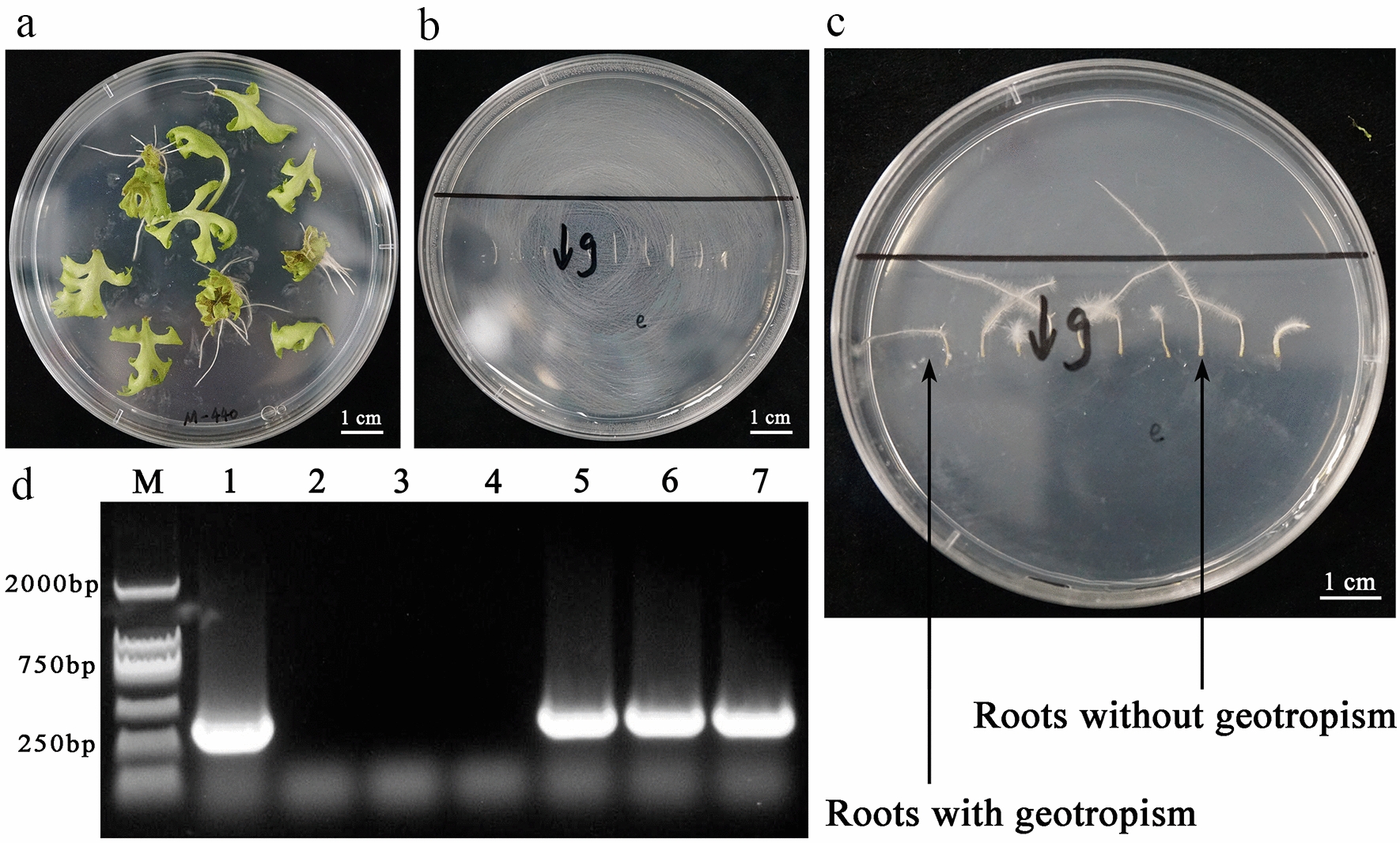
Induction of pyrethrum hairy roots. **a** Twenty days after transformation, roots emerged from the infected leaf explants. **b** Root tips were placed on culture medium and culture dishes oriented vertically to ensure that the root tips were upward. **c** After a week of cultivation, two different root types appeared: one exhibiting geotropism and the other without geotropism. **d** PCR detection of *rol B* in the two types of root. M, marker; 1, bacterial plasmid DNA control; 2–4, DNA from roots exhibiting geotropism; 5–7, DNA from roots without geotropism. Scale bars in **a**–**c**, 1.0 cm

Leaves from pyrethrum tissue culture plantlets were used as explants, infected with *A. rhizogenes*, and roots appeared on explants after 20 days (Fig. 1a). Root tips were excised and placed on culture medium with the root tip upward (Fig. 1b). When the root tips were placed upside down for 7 days, two kinds of roots were identified: one type exhibiting geotropism and another without geotropism (Fig. 1c). Specific primers for the *rol B* gene on the Ri plasmid were used for PCR amplification of DNA extracted from the two types of root as template, and roots without geotropism were identified as containing the *rol B* gene (Fig. 1). Therefore, in the present study, geotropism was used to select hairy roots carrying the Ri plasmid.

**Fig. 2:**
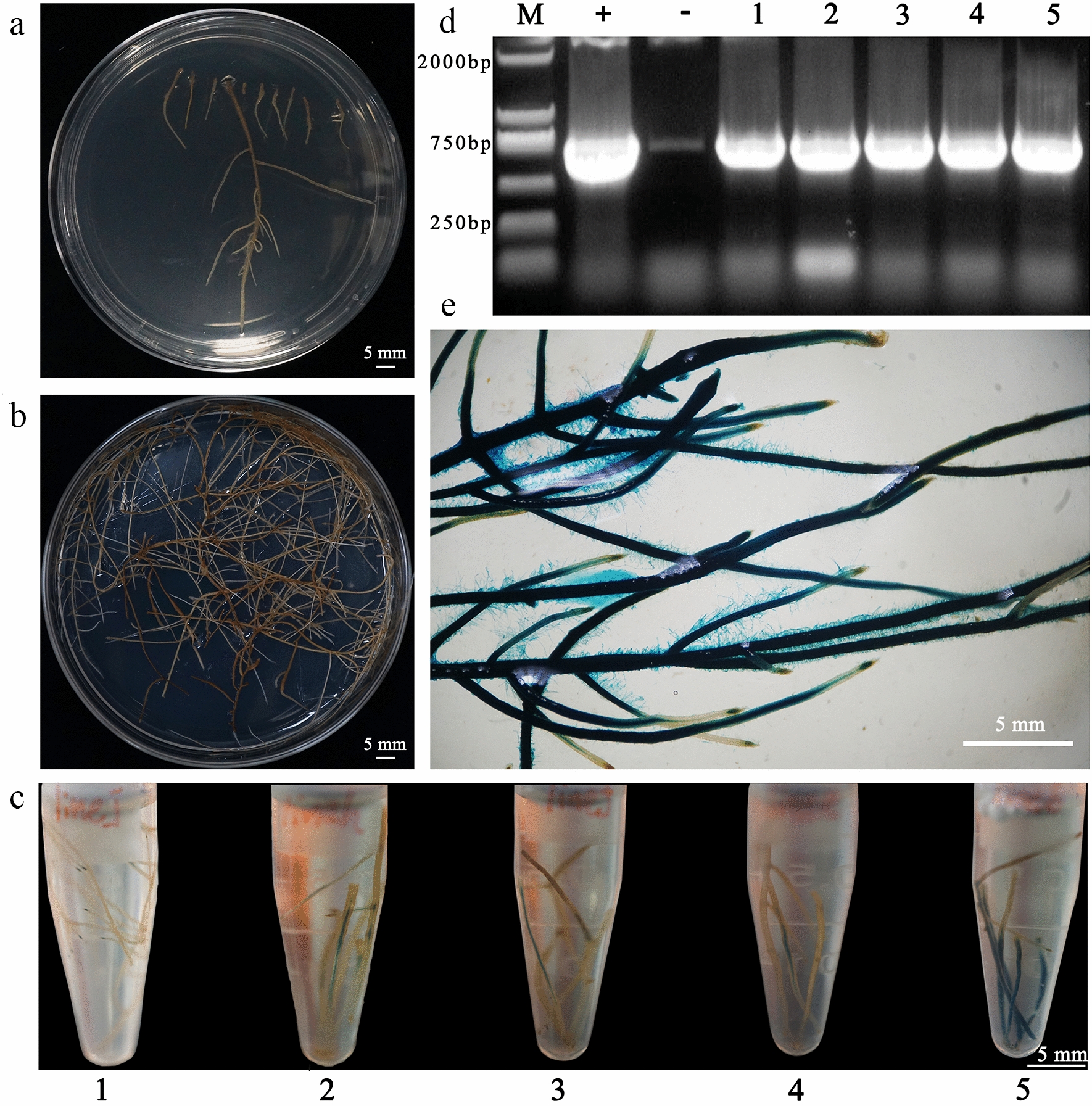
Transformation of pyrethrum mediated by *A. rhizogenes* harboring the pBI121 vector. **a** Resistant roots grown on selective medium containing kanamycin (10 mg/L) for 20 days. **b** Resistant hairy roots were excised and transferred onto selective medium containing kanamycin (10 mg/L) to propagate for approximately 60 days. **c** Transgenic roots stained with X-gluc reagent. **d** PCR detection of *NPT II* in samples from 60-day-old resistant roots. M, marker; +, bacterial plasmid DNA control; −, wild type plant control. **e** Magnified image of dyed roots from Line 5. Numbers represent different lines, as follows: 1, Line 1; 2, Line 2; 3, Line 3; 4, Line 4; 5, Line 5. Scale bars in** a**–**c** and **e**, 5.0 mm

After screening for roots without geotropism, root tips were cut off and placed in kanamycin screening medium to select hairy roots carrying Ti plasmids (Fig. 2a). The resulting proportion of explants inducing roots was 51% ± 4% (100 explants, four replicate experiments), that of roots without geotropism was approximately 26% (10 roots, five replicate experiments), and the mean proportion of resistant roots was 7% (50 roots, four replicate experiments). Next, resistant roots were cut into 1-cm-long segments and inoculated in the same medium. These resistant roots continued to grow and could grow lateral roots (Fig. 2b). To further determine whether T-DNA was transferred into the hairy roots, resistant roots were stained with X-gluc reagent and Lines 1–5 were dyed blue (Fig. 2c). Next, DNA samples were extracted from these roots for PCR analysis. All roots were found to contain the *NPTII* gene fragment from the pBI121 vector (Fig. 2d). These results indicated that hairy roots stably transformed with pBI121 could be obtained via geotropism and kanamycin screening, and that the *CaMV35S* promoter was suitable for high level foreign gene expression in pyrethrum hairy roots.

### Analysis of gene editing in transgenic hairy roots

**Fig. 3:**
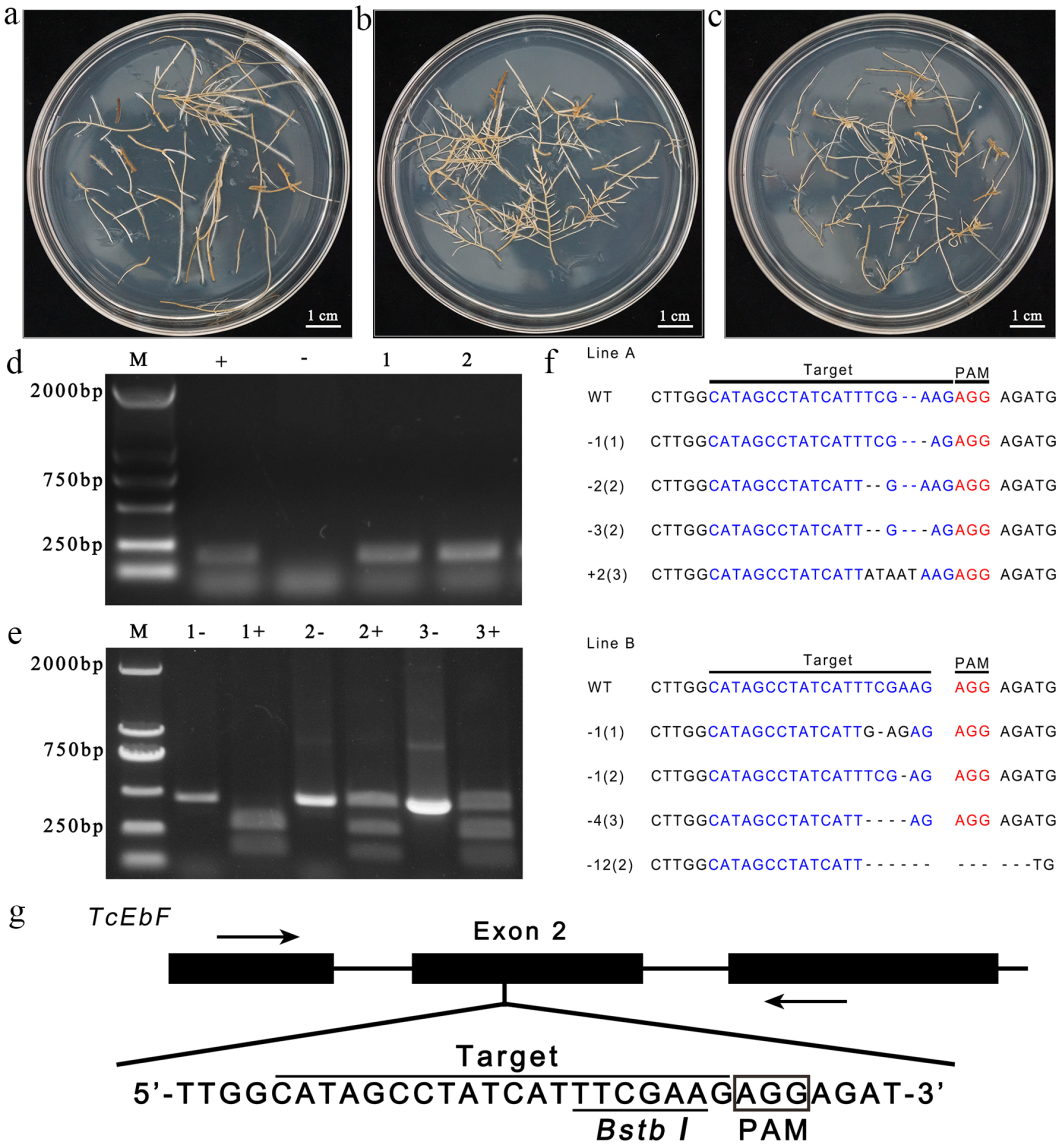
Analysis of gene editing of transgenic hairy roots 1 month after subculture. **a** Control group (CK). **b** Line A. **c** Line B. **d** Detection of the *RGR* sequence by PCR. M, marker; +, ribozyme-based CRISPR/Cas9 vector; −, CK; 1, line A; 2, line B. **e** PCR amplification followed by restriction enzyme digestion detection of target sites: M, marker; 1−, CK *TcEbFS* amplicon; 1+, CK *TcEbFS* fragments following digestion with by *BstbI*; 2−, Line A *TcEbFS* amplicon; 2+, Line A *TcEbFS* fragments following digestion with *BstbI*; 3−, Line B *TcEbFS* amplicon; 3+, Line B *TcEbFS* fragments following digestion with *BstbI*. **f** DNA sequencing confirming *TcEbFS* gene mutations in pyrethrum hairy roots. The net length of insertions and/or deletions are presented in the column to the left. Numbers in parentheses indicate the number of clones. **g** Diagram showing the *TcEbFS* genomic locus with the sgRNA targeting site and primer locations (arrows). Scale bars in **a**–**c**, 1.0 cm

The control group (CK) hairy root line (Fig. 3a), in which the *CaMV35S* promoter drives *GUS*; and the resistant hairy root lines, line A (Fig. 3b) and line B (Fig. 3c), in which the *CaMV35S* promoter drives *RGR* and *Cas9*, were obtained. DNA was extracted from resistant roots, and PCR analysis showed that an *RGR* fragment of approximately 229 bp was amplified from the positive control, as well as lines A and B, whereas the amplicon was not detected in CK hairy root samples. These results confirm that lines A and B were transgenic roots carrying the CRISPR/Cas9 vector (Fig. 3d). Furthermore, PCR amplification followed by restriction enzyme digestion was used to detect editing of target sites in lines A and B. In the control group, two fragments of 162 and 272 bp were produced after the complete digestion by *BstbI*, while the *TcEbFS* gene fragments amplified from lines A and B were not completely cleaved by *BstbI* (Fig. 3e). These findings indicated that no *TcEbFS* gene fragments in the CK group were edited and contained the *BstbI* restriction site (TT/CGAA), while the *TcEbFS* gene fragments from lines A and B contained edited *TcEbFS* gene fragments, in which the restriction site was destroyed. The *TcEbFS* gene fragment amplified from the CK group, and the *BstbI* resistant *TcEbFS* gene fragments from lines A and B were then sequenced. The results showed that the *TcEbFS* sequence of the CK group was identical to the published *TcEbFS* sequence (MF682058), whereas the *BstbI*-resistant *TcEbFS* gene fragments from lines A and B carried mutations, including nucleotide insertions and deletions causing amino acid deletions and frame shifts (Fig. 3f).

### (E)-β-farnesene content in hairy roots

**Fig. 4:**
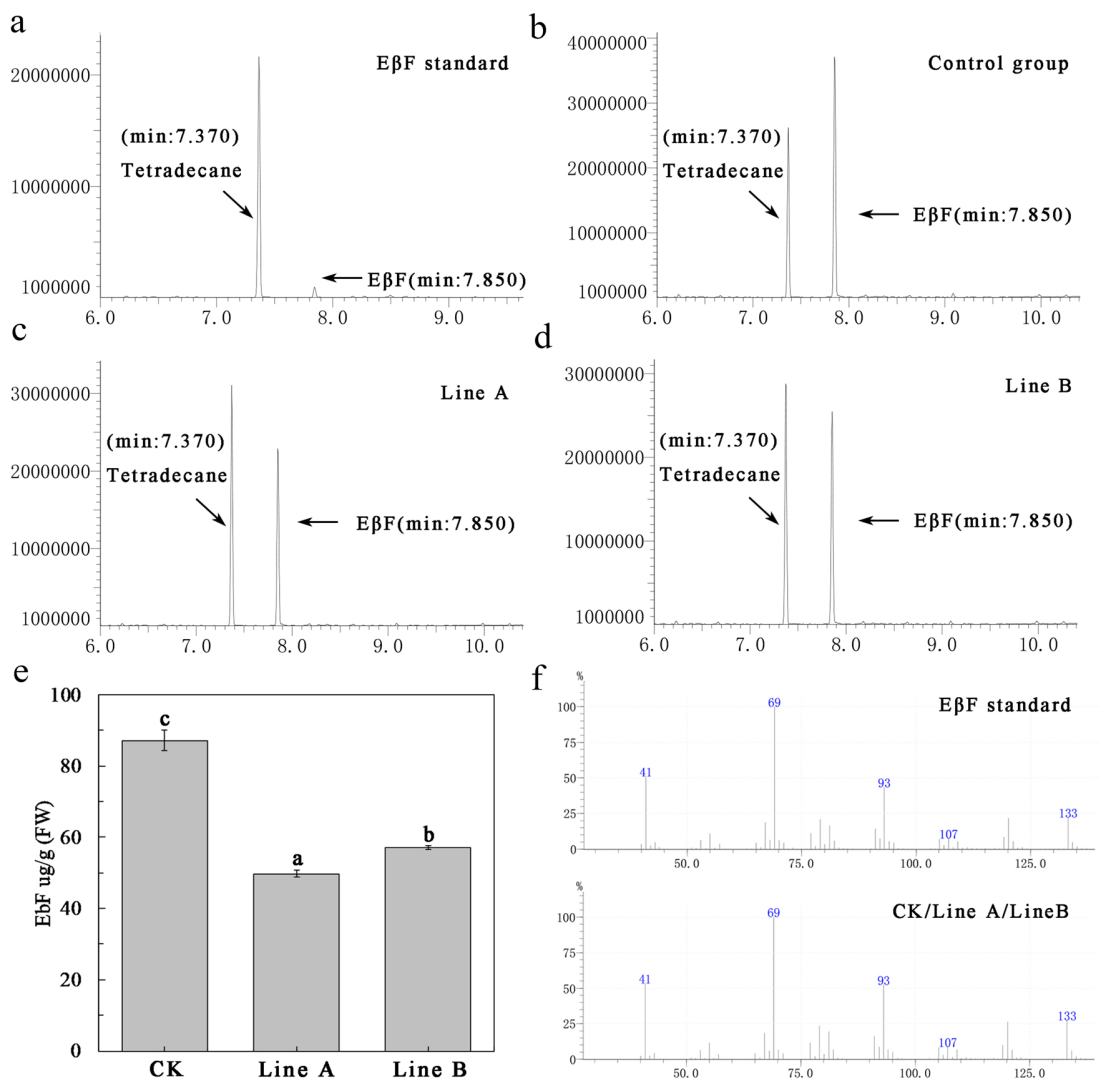
(E)-β-farnesene content in hairy roots. **a** (E)-β-farnesene standard (1 ng/µl). **b** Control group (CK). **c** Line A. **d** Line B. **e** (E)-β-farnesene content in CK, line A, and line B. **f** 7.850 min mass spectra

The (E)-β-farnesene content of CK, line A, and line B was determined by GC–MS. That of the CK group was 87.19 ± 2.89 µg/g (fresh weight (FW)) (Fig. 4b), that of line A was 49.77 ± 1.05 µg/g (FW) (Fig. 4c), and that of the line B was 57.13 ± 0.52 µg/g (FW) (Fig. 4d). These results showed that pyrethrum hairy roots’ (E)-β-farnesene content was very high, being significantly lower in lines A and B s than the CK group (Fig. 4e).

## Discussion

### A rapid and highly efficient *A. rhizogenes-*mediated hairy root transformation system for pyrethrum

To date, there has been only one report of pyrethrum transformation using an *A. tumefaciens*-mediated system based on leaf explants; however, such methods are unsuitable for functional verification of novel CRISPR/Cas9 systems, because they are time-consuming and extremely inefficient [30]. Therefore, in the present study a new pyrethrum transformation method for the rapid verification of novel CRISPR/Cas9 systems was established. It achieved a high hairy root transformation frequency (7%) and the process took only 49 days (2 days of co-culture, 20 days of root formation, 7 days of screening for roots without geotropism, and 20 days of screening for kanamycin-resistant roots).

Transgenic hairy roots are transformed by pRi and pTi [31]; therefore, both plasmids’ characteristics were used to screen for transgenic hairy roots. In a study of *Brassica napus*, pRi-transformed roots were less sensitive to gravity than normal roots [32]. This phenomenon of reduced geotropism was also reported in sweet potato hairy roots [33] and is caused by *aux* genes carried on pRi that interfere with hairy root gravitropism [34]. Therefore, this characteristic loss of gravitropism was employed here to screen for hairy roots transformed by pRi. Kanamycin resistance was used to screen for transgenic hairy roots transformed by pTi. Due to the co-transformation of pRi and pTi, transgenic hairy roots are chimeras (Additional file 1: Fig. S1). This phenomenon has also been reported in *Arachis hypogaea*, *Taraxacum hybernum*, and *Althaea officinalis* [35–37]. Nevertheless, this method is suitable only for qualitative, but not for quantitative, research; hence, gene editing efficiency could not be determined in the later experiments.

### Advantages of the ribozyme-mediated CRISPR/Cas9 system for pyrethrum research

High expression of sgRNA in cells is critical to the success of gene editing [38]. In general, sgRNA is expressed from the *U6* or *U3* promoter in the CRISPR/Cas9 system; however, as in most plants, no *U6* or *U3* promoters have been identified in pyrethrum, making it difficult to select a functional *U6* or *U3* promoter for use in CRISPR/Cas9 systems [17]. Therefore, gene editing methods using a pol II promoter were sought. Post-transcriptional modification of RNA at both ends influences the ability of an sgRNA to guide Cas9 to cut a target site [39, 40]. Hence, separation of the modified structures at both ends of an sgRNA and conversion of the mRNA into sgRNA is desirable. Using the characteristic of ribozyme self-cleavage, an sgRNA with editing ability can be separated from the cap and tail structures of mRNA. This method is referred to as the *RGR* strategy and was first successfully applied to yeast [17], and later to zebrafish (*Danio Rerio*) [41], human cells [42], mice [43], *Plasmodium yoelii* [44], *Arabidopsis thaliana* [45], and *Oryza sativa* [46]. Moreover, some pol II promoters are more efficient than the *U6* or *U3* promoter [47]. The results reported here are similar to those of other studies, suggesting the *RGR* strategy is generally applicable to eukaryotes. Applying the *RGR* strategy reduces the difficulty of promoter selection for use in pyrethrum gene editing.

In addition to the easy availability of the promoter, ribozyme-based CRISPR/Cas9 technology has other advantages. *U6* and *U3* promoters mediate constitutive expression in plants, and the CRISPR/Cas9 technology that relies on *U6* or *U3* to initiate sgRNA transcription cannot be used for specific editing [46]. The *RGR* strategy increases the types of promoters, allowing CRISPR/Cas9 system to use a wide variety of pol II promoters besides *U6* and *U3* promoters [17]. Therefore, this technology could greatly promote pyrethrum’s gene function research. For example, pyrethrum has three types of (E)-β-farnesene with reported differences in release mode, as follows: large release after leaf damage, large release during flower development, and sustained and stable low-level release [23, 24]. The (E)-β-farnesene released in these three modes can interfere with one another, rendering it challenging to study the biological significance of (E)-β-farnesene in each mode. The best way to investigate the particular biological functions of these types of (E)-β-farnesene is to silence each separately, and applying the *RGR* strategy could theoretically realize such tissue-specific gene editing [17].

This technology is also promising for breeding of pyrethrum disease resistance. The most damaging foliar disease of pyrethrum is ray blight caused by *Stagonosporopsis tanaceti* [48]. The emergence of CRISPR/Cas9 technology provides a new approach to address this problem, and this technology has worked to achieve resistance to pathogens in various crops [49–51]; however, to reduce the negative effects of a transgene on the whole plant, the most efficient way to confer resistance is to restrict its expression only to infected cells. Pathogen-induced promoters are unique and valuable tools for engineering resistance to plant disease [52, 53]. Therefore, integration of the CRISPR/Cas9 system and pathogen-induced promoters (pol II promoter) to develop a pathogen-inducible gene editing system is of great significance for devising pyrethrum resistance to *S. tanaceti*, as this method can restrict gene editing only to infected cells and reduce metabolic disruption caused by gene editing, thus minimizing the impact on plant growth and development.

## Conclusions

This work established a hairy root transformation system mediated by *A. rhizogenes* in pyrethrum. Using this system, a self-cleaving ribozyme-mediated genome editing method using the *CaMV35S* promoter was evaluated. This system increased the types of promoters capable of transcribing sgRNA and can potentially be expanded to other RNA polymerase II-dependent promoters with strong capacity for expression in specific tissues or under certain conditions, to achieve site-specific gene editing in pyrethrum.

## Methods

### Plant material

Pyrethrum clone W99 was obtained from the Key Laboratory for Biology of Horticultural Plants, Ministry of Education, College of Horticulture & Forestry Sciences, Huazhong Agricultural University. Clones were inoculated on Murashige and Skoog (MS) solid medium for 30 days after subculture, and their leaves used for transformation. Cultures were maintained at 25 °C ± 2 °C, with a photoperiod of 16/8 h at light intensity 40 μmol m^−2^ s^−1^.

### Construction of ribozyme-based CRISPR/Cas9 vectors

**Fig. 5:**
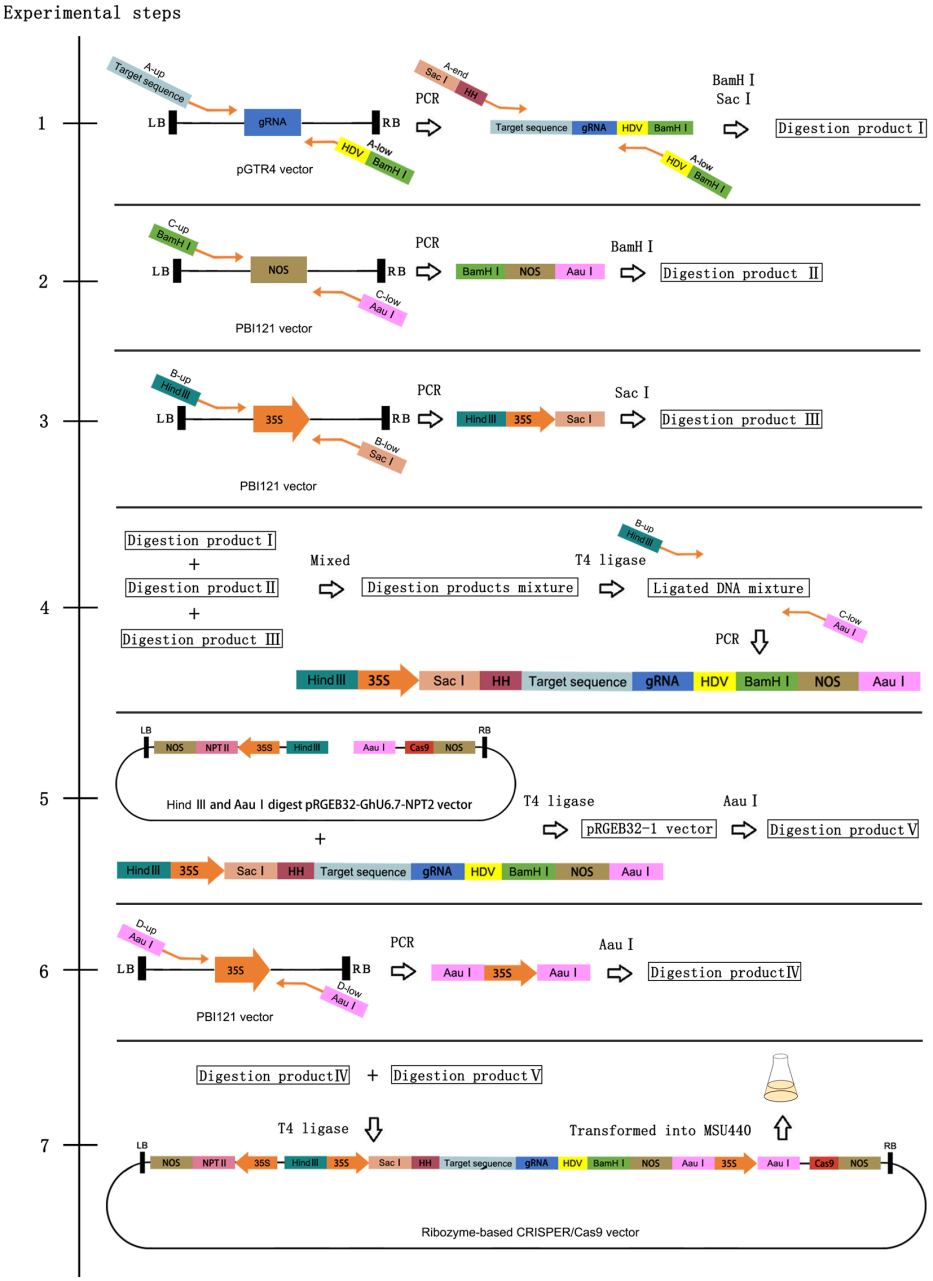
Ribozyme-based CRISPR/Cas9 vector construction process. The hammerhead-type ribozyme sequence is labeled HH, and the hepatitis delta virus ribozyme sequence is labeled HDV. Step 1: The 229-bp fragment encompassing the ‘SacI + HH + sgRNA + HDV + BamHI’ fragment was obtained. Next, this fragment was digested by SacI and BamHI. Step 2: The 288-bp fragment encompassing the ‘BamHI + NOS + Aaul’ fragment was obtained. Next, this fragment was digested by BamHI. Step 3: The 853-bp fragment encompassing the ‘HindIII + 35S + SacI’ fragment was obtained. Next, this fragment was digested by SacI. Step 4: Their digestion products were mixed at the same molar concentration and ligated. Next, using the ligated DNA mixture as a template, a 1346-bp fragment encompassing the ‘HindIII + 35S + HH + sgRNA + HDV + NOS + Aaul’ was obtained. Step 5: The pRGEB32-GhU6.7-NPT2 vector and the ‘HindIII + 35S + HH + sgRNA + HDV + NOS + Aaul’ fragment were ligated. Next, the plasmid was digested by Aaul. Step 6: The 853 bp fragment ‘Aaul + 35S + Aaul’ was obtained. Next, this fragment was digested by Aaul. Step 7: The digested plasmid in step 5 and the digested fragment ‘Aaul + 35S + Aaul’ were connected

According to Liang et al.’s methods [54], a target site with a PAM sequence in exon 2 of the *TcEbFS* gene was screened (Fig. 3g). The target site contained a *BstBI* (TT/CGA) restriction site, which can be used for an editing analysis by the restriction enzyme site-loss method [55]. The plasmids, pRGEB32-GhU6.7-NPT2 and pGTR4, were gifted from the National Key Laboratory of Crop Genetic Improvement, Huazhong Agricultural University [56]. Ribozyme-based CRISPR/Cas9 vectors were constructed using the *TcEbFS* gene target sequence, based on pRGEB32-GhU6.7-NPT2. The process of vector construction is illustrated in Fig. 5. The primers used in the experiment are listed in Additional file 2: Table S1. A plasmid with the correct sequence was transferred into *A. rhizogenes* MSU440.

### Establishment of a stable hairy root transformation system

Leaves were excised from pyrethrum clone W99 and infected with *A. rhizogenes* MSU440 containing the pBI121 vector. The process of *Agrobacterium* infection is now well described in the literature [30]. First, hairy roots stably transformed with Ri plasmids were screened. Then, infected leaves were placed on solid 1/2 MS medium containing 400 mg/L cefotaxime. After 20 days of cultivation in the dark, roots appeared in the pyrethrum leaves and root tips were excised, placed horizontally on culture medium, and culture dishes oriented vertically, to ensure that the root tip was pointing upward. After 1 week of cultivation in the dark, two different hairy roots appeared: those exhibiting geotropism and those showing no geotropism. DNA was extracted from these two different types of root, and three agrobacterium-free samples extracted from each type of root. PCR-based amplification of *vir* genes was used to test for agrobacterium contamination [57, 58]; primers specific for the *TetR* gene were used, where detection of *TetR* indicated agrobacterium contamination and the sample was discarded.

Uncontaminated samples were used to analyze the *rol B* gene. Specific primers, rol B-up and rol B-low, were designed based on the *rol B* gene sequence of the *A. rhizogenes*’ Ri plasmid, according to the description by Xiao et al. [59]; theoretically, these primers amplify a 450-bp gene fragment from hairy roots stably transformed with Ri plasmids. Next, hairy roots stably transformed with Ti plasmids were screened. Roots without geotropism were placed horizontally in 1/2 MS medium containing kanamycin (10 mg/L) and cefotaxime (400 mg/L). After 20 days in the dark, numbers of elongated roots were counted. Kanamycin-resistant roots were inoculated in 1/2 MS medium containing kanamycin (10 mg/L) and cefotaxime (400 mg/L), and sub-cultured every 60 days. Kanamycin-resistant roots were histochemically stained using the beta-glucuronidase (GUS) Staining Kit (Coolaber, China) to determine the stability of the transformation and visualize transgene expression.

**Fig. 6:**
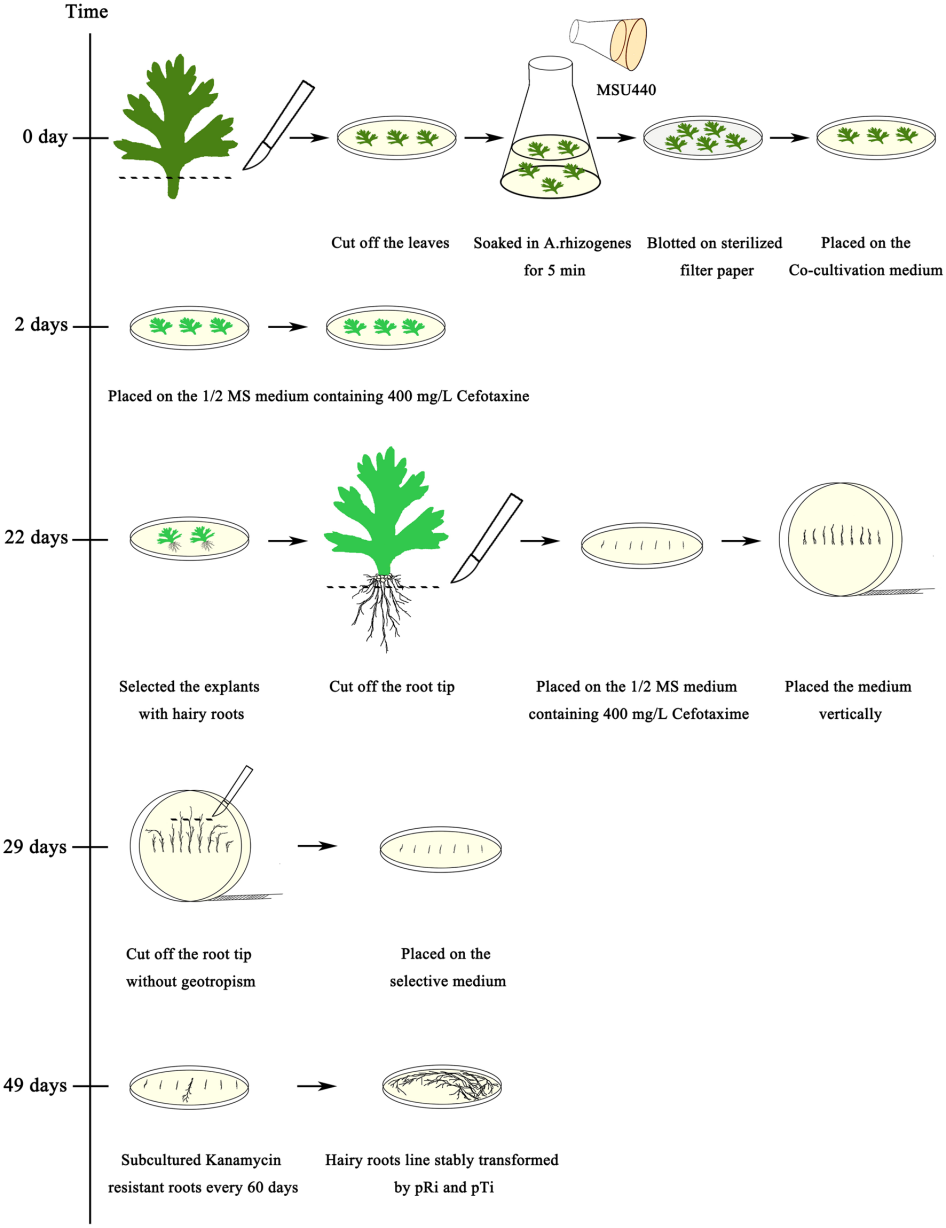
Pyrethrum hairy root transformation workflow using leaf explants. At 0 days: *Agrobacterium* infection. *Agrobacterium* cell density with an OD_600_ = 0.5. Co-cultivation began. At 2 days: Co-cultivation was over. Roots’ induction began. At 22 days: Roots’ induction was over. Screening of roots transformed with Ri began. At 29 days: Screening of roots transformed with Ri was over. Screening of roots transformed with Ti began. At 49 days: Screening of roots transformed with Ti was over. Sub-cultivation began

The specific primers, NPTII-up and NPTII-low, were designed based on the pBI121 plasmid *NPTII* gene sequence. DNA from kanamycin resistant roots served as a template for the PCR analysis. Theoretically, a 750-bp gene fragment could be amplified from hairy roots stably transformed with the pBI121 vector. Introduction of the *TcEbFS* ribozyme-based CRISPR/Cas9 vector was conducted under the same conditions (Fig. 6). To confirm the presence of transgenes in putatively transformed hairy roots, HH-sgRNA-HDV sequence specific primers (A-end and A-low) were designed. A PCR product of 229 bp was expected from positive hairy roots, and positive samples according to the PCR analysis were used for subsequent experiments.

### Detection of *TcEbFS* editing

A *TcEbFS* gene fragment (approximately 456 bp) was amplified using specific primers (F-up and F-low) across the editing site. As the fragment contained the TT/CGAA restriction site, the *TcEbFS* gene amplification product from the control group could be recognized and completely digested into 162-bp and 272-bp fragments by *BstbI*; however, amplified *TcEbFS* gene products from edited transgenic hairy roots contained fragments could not be recognized and cleaved by *BstbI*. Undigested bands were ligated into the pCloneEZ vector, and the vector was transferred into *Escherichia coli* DH5α. Clones were randomly selected and sequenced to detect gene mutations, and eight clones were selected from each line. Hairy roots transformed with the pBI121 vector alone served as the control.

### Detection of (E)-β-farnesene in gene edited hairy roots

Gas chromatography-mass spectrometry (GC–MS) was used to analyze secondary metabolites of hairy root lines containing the ribozyme-based CRISPR/Cas9 vector grown on 1/2 MS medium for 60 days. After freezing in liquid nitrogen and grinding using a mortar and pestle, hairy roots (500 mg) were transferred into a 5-mL tube, and 1 mL of methyl-tert-butyl ether (MTBE) containing tetradecane (0.01 ng/mL) added as an internal standard. The tube was vortexed for 3 min at maximum speed, incubated at 24 °C with a rotation speed of 50 rpm, then dried using Na_2_SO_4_. For GC–MS analysis, 1-µL sample aliquots were injected into a GC/MS-QP 2010 Ultra instrument (Shimadzu Corporation, Japan) with an HP-5 MS column. The relative concentration of (E)-β-farnesene was calculated by the area ratio of the (E)-β-farnesene peak to that of the internal standard.

Hairy roots transformed with the pBI121 vector were used as a control. Helium (1.4 mL/min) was used as a carrier gas. The injection temperature was set at 240 °C. The oven temperature program went as follows: initial temperature 50 °C, followed by a ramp from 50 to 150 °C at a rate of 20 °C/min, held for 1 min, and then from 150 to 180 °C at 20 °C/min, held for 1 min, and finally increased to 300 °C at 30 °C/min, held for 5 min. Identification of volatiles was conducted by comparing their retention times and mass fragmentation values with those reported in the literature, and the NIST (2017) and PESTEI_3.lib databases. Spectral data were also compared to (E)-β-farnesene standards (Sigma, Germany), these diluted to 1 ng/μL with MTBE.

### Statistical analysis

Data obtained from all experiments are presented as means ± standard error, and separations performed using the least significant difference (LSD) test. Percentage data were subjected to angular transformation and evaluated by ANOVA using SPSS software v17.0. Values with different letters show significant differences at P ≤ 0.05 (LSD).

## Supplementary Information

**Additional file 1: Fig. S1.** Transgenic roots (pBI121) stained with x-gluc reagent showing transgenic hairy root chimeras.

**Additional file 2: Table S1.** Primers and construction process of ribozyme-based CRISPR/Cas9 vectors used in the experiment.

## Data Availability

The datasets generated and analyzed in the current study are available from the corresponding author on reasonable request.
