# Supplementary material for: Ribozyme-mediated CRISPR/Cas9 gene editing in pyrethrum (Tanacetum cinerariifolium) hairy roots using a RNA polymerase II-dependent promoter
Source: Plant Methods. 2022 Mar 16;18:32. doi: 10.1186/s13007-022-00863-5 (PMC8925089; doi:10.1186/s13007-022-00863-5)
Supplement: Supplementary file 1 — Additional file 1: Fig. S1. Transgenic roots (pBI121) stained with x-gluc reagent showing transgenic hairy root chimeras. [file 13007_2022_863_MOESM1_ESM.docx]

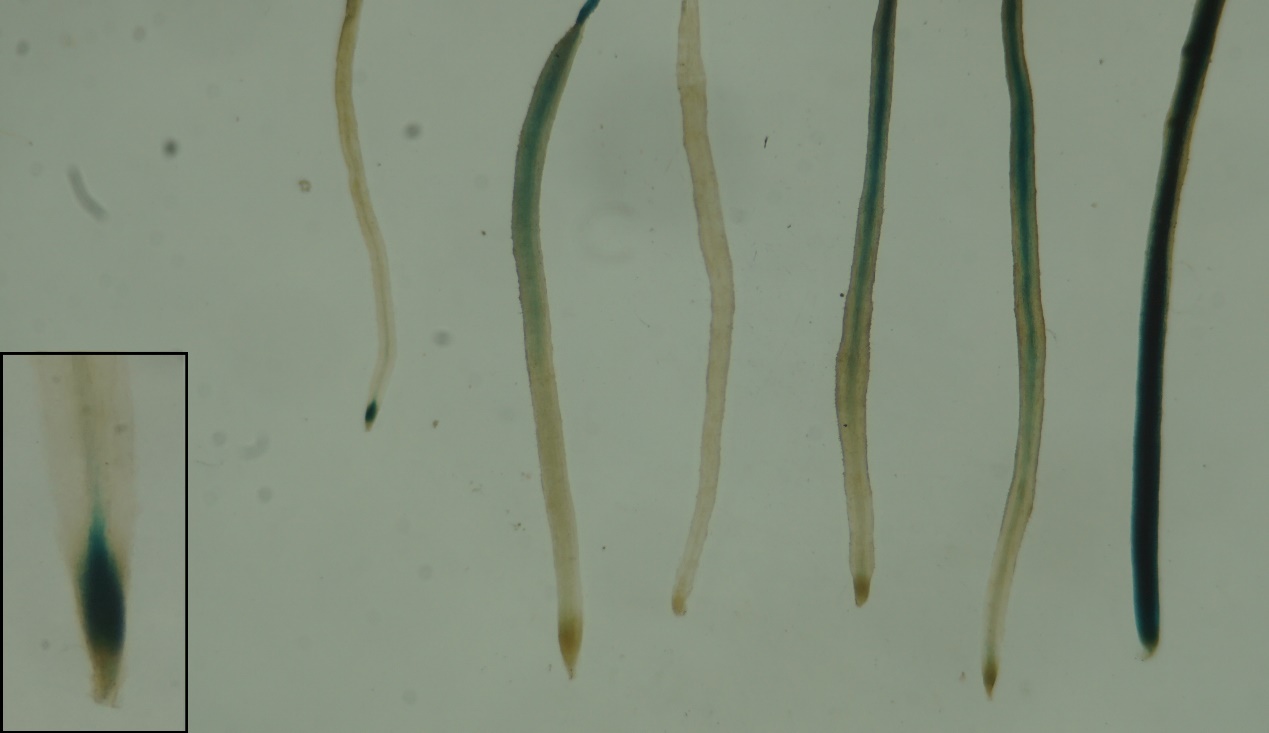


Fig S1. The transgenic roots (pBI121) were stained with an x-gluc reagent and the transgenic hairy roots are chimeras.
