# Supplementary material for: Ribozyme-mediated CRISPR/Cas9 gene editing in pyrethrum (Tanacetum cinerariifolium) hairy roots using a RNA polymerase II-dependent promoter
Source: Plant Methods. 2022 Mar 16;18:32. doi: 10.1186/s13007-022-00863-5 (PMC8925089; doi:10.1186/s13007-022-00863-5)
Supplement: Supplementary file 2 — Additional file 2: Table S1. Primers and construction process of ribozyme-based CRISPR/Cas9 vectors used in the experiment. [file 13007_2022_863_MOESM2_ESM.docx]

Table S1 Primers and construction process of ribozyme-based CRISPR/Cas9 vectors used in the experiment

| Primers | Sequence (5’-3’) |
| --- | --- |
| A-up | CATAGCCTATCATTTCGAAGGTTTTAGAGCTAGAAATAG |
| A-low | TATGGATCCGTCCCATTCGCCATGCCGAAGCATGTTGCCCAGCCGGCGCCAGCGAGGAGGCTGGGACCATGCCGGCCAAAAGCACCGACTCGGTGCC |
| A-end | ATAGAGCTCTTCCACCTGATGAGTCCGTGAGGACGAAACGAGTAAGCTCGTCCATAGCCTATCATTTCGAAG |
| B-up | CGCAAGCTTAGATTAGCCTTTTCAATTTC |
| B-low | TATGAGCTCCGTGTTCTCTCCAAATG |
| C-up | ATAGGATCCGGATGATCCCCGATCGTTC |
| C-low | CGCTGTACAAGGCCCGATCTAGTAAC |
| D-up | CGCTGTACAAGATTAGCCTTTTCAATTTC |
| D-low | TCGTGTACACGTGTTCTCTCCAAATG |
| E-up | GATAAATTATCGCGCGCGGTGTCATCTATG |
| E-low | CGACCTTCCGCTTCTTCTTTGGG |
| F-up | GCAGCCAGAAGACTTGGTTACGAAG |
| F-low | AGTCCTTGAATACTCCTGTAGAATG |
| rol B-up | GCTCTTGCAGTGCTAGATTT |
| rol B-low | GAAGGTGCAAGCTACCTCTC |
| NPTII-up | AGAGGCTATTCGGCTATGACTG |
| NPTII-low | GCTCAGAAGAACTCGTCAAGAAG |
